# Neurotrophic Factors: Emerging Biology and Therapeutic Applications for Cardiovascular Diseases

**DOI:** 10.3390/metabo16010058

**Published:** 2026-01-09

**Authors:** Yu Liu, Huijie Zhang, Fengzhi Yu, Tiemin Liu, Dandan Jia, Ruwen Wang

**Affiliations:** 1 School of Exercise and Health, Shanghai University of Sport, 188 Hengren Road, Yangpu District, Shanghai 200438, China; 2 State Key Laboratory of Genetics and Development of Complex Phenotypes, Fudan University, Shanghai 200438, China

**Keywords:** cardiovascular diseases, neurotrophic factors, neurocardiac interactions, exercise, 7,8,3′-Trihydroxyflavone, 7,8-Dihydroxyflavone

## Abstract

Cardiovascular diseases (CVDs) have emerged as a common health problem. However, despite their prevalence, little progress has been made in their treatment. In recent years, neurotrophic factors (NTFs) have been discovered to exert cardioprotective functions for CVDs. NTFs can modulate vascular integrity, myocardial remodeling, angiogenesis, and autonomic regulation, playing the roles of maintaining cardiovascular homeostasis and influencing disease progression. Under pathological conditions, the supplement of NTFs can induce substantial adaptations to mitigate adverse cardiac responses. Several NTFs have been investigated in this regard. This review briefly elaborates on present insights into the expression, signaling pathways, and regulatory effects of NTFs on the development of CVDs, and also discusses emerging therapeutic strategies based on NTFs, ranging from exercise to advanced modalities including stem cell therapy, gene transfer, recombinant protein therapy and NTF mimetics, among which the mimetics and exercise interventions emerge as the most promising avenues for clinical translation.

## 1. Introduction

Cardiovascular diseases (CVDs) are one of the predominant contributors to worldwide disease burden and mortality, becoming a critical and ongoing global health priority [1]. Projections reveal a substantial upward trajectory in the global burden of CVDs from 2025 to 2050. Over this period, the prevalence of CVDs is anticipated to rise by 90.0%, while crude mortality and crude disability-adjusted life years are, respectively, projected to increase by 73.4% and 54.7%. As a result, the annual number of cardiovascular deaths is expected to rise from 20.5 million in 2025 to 35.6 million by 2050 [2]. Although dietary and lifestyle interventions can effectively reduce the prevalence and mortality, there are low rates of long-term successes [3,4]. Therefore, many drugs have been developed for treating CVDs. However, pharmacotherapy is usually accompanied by a change in patients’ status (e.g., pregnancy, cost, side effects), which might finally not be sufficient to correct clinical symptoms for some individuals [5]. Therefore, an urgent need exists for novel cardiovascular therapeutics.

Neurotrophic factors (NTFs) are secreted proteins derived from nervous tissues that regulate neurite outgrowth, neuronal differentiation, survival, and communication between neurons and their target tissues [6]. They include primarily the neurotrophin (NT) family, the glial-derived neurotrophic factor (GDNF) family, ciliary neurotrophic factor (CNTF), and the recently identified cerebral dopamine neurotrophic factor (CDNF)/mesencephalic astrocyte-derived neurotrophic factor (MANF) family [7,8]. Like the brain, several tissues also abundantly express NTFs, including heart, liver, pancreas, kidneys, and skeletal muscles and intestine, which can explain why NTFs can modulate the function of non-neuronal tissues through autocrine and paracrine mechanisms [9,10,11]. Levels of NTFs markedly change in humans during cellular stress, such as in neurodegenerative diseases, metabolic syndromes, cancers, immune diseases and CVDs. Significant alterations in NTF levels can indicate adverse responses to cardiac rehabilitation in patients with CVDs [12]. NTFs contribute to cardiac remodeling, regulation of angiogenesis, cardiomyocyte contractility, and maintenance of overall cardiac integrity in the cardiovascular system [13]. In particular, neurotrophin signaling through the tropomyosin receptor kinase (Trk) family receptors is indispensable for the development of embryonic cardiomyocytes, as demonstrated by severe cardiac defects observed in mice lacking neurotrophins or Trk family receptors. However, the involvement of NTFs with heterogeneous sympathetic innervation might increase susceptibility to ventricular arrhythmias and sudden cardiac death. Therefore, NTFs represent promising therapeutic targets for cardiac conditions.

In this review, we highlight NTFs’ production and secretion as well as what is known about their signaling. We also synthesize current preclinical and clinical findings providing evidence that NTFs might be an effective drug target. The review will further critically evaluate the therapeutic strategies that have been applied, including their current progress and existing challenges. Finally, we summarize and critically analyze the roles of various NTFs in the progression of CVDs in both animal models and humans.

## 2. Biological Functions of Neurotrophic Factors

The nerve growth factor (NGF) family of growth factors, collectively known as neurotrophins (NTs), regulates extensive biological functions and was initially recognized for its roles in neuronal survival, growth, and plasticity. Conventional NT family members comprise brain-derived neurotrophic factor (BDNF), NGF, NT-3, and NT-4/5, with NT-6 and NT-7 later identified as novel neurotrophins [14]. A hallmark of NTs is their initial synthesis as a precursor protein that must be proteolytically processed, either intracellularly or in the extracellular environment. Current evidence indicates that the NT-6 and NT-7 genes are restricted to fish lineages, with no definite homologs in mammalian or avian species [15]. NTs exert their effects through two distinct receptor types: the receptors of the Trk family and the p75 neurotrophin receptor (p75^NTR^). Owing to the differences in binding affinity and microenvironment, ligand–receptor interactions display distinct specificities: NGF selectively activates TrkA, whereas BDNF and NT-4/5 bind to TrkB. NT-3 primarily engages TrkC with high affinity but also stimulates TrkA and TrkB, albeit with reduced efficiency [16]. In contrast, p75^NTR^ interacts with all NTs, though with relatively low affinity [16].

GDNF, neurturin, persephin, and artemin together form the GDNF family. Although these proteins exhibit limited amino acid sequence homology, they all function as homodimers to activate the receptor tyrosine kinase rearranged during transfection (RET). Similarly to NTs, the GDNF family signals through two receptor systems: RET and GDNF family receptor alpha (GFRa), which are glycosylphos-phoinositol (GPI)-linked receptors [17,18]. Specifically, GDNF signals through GFRa1, NRTN through GFRa2, ARTN via GFRa3, and PSPN through GFRa4 [19]. Notably, GFRa2 has been shown to regulate cardiomyocyte differentiation through a RET-independent signaling pathway [20]. Although all co-receptors are capable of interacting with RET, ligand specificity is largely determined by their differential binding affinities. Cross-signaling has been demonstrated. For instance, GDNF has the ability to activate GFRa2, while NRTN utilizes GFRa1 [21]. Furthermore, GPI-anchored co-receptors, while initially membrane-bound, can be cleaved and subsequently function as soluble co-receptors, thereby extending their role in signaling. Consequently, when soluble co-receptors are present, GDNF family ligands retain the capacity to activate RET even in cells that do not naturally express these co-receptors. GDNF is initially synthesized as a precursor protein and subsequently processed into its active form through sequential cleavage events [22]. By binding to its receptors, active GDNF triggers multiple downstream pathways controlling neuronal development, survival, and neuron–tissue communication [23]. In addition, akin to MANF/CDNF, GDNF can modulate endoplasmic reticulum (ER) stress and mitigate protein aggregation and inflammation [24]. Dysregulation of GDNF synthesis and signaling has been reported in a variety of pathological conditions, as well as in the contexts of aging, exercise, and addiction [25,26].

Originally identified in chick embryo ciliary neuron, CNTF possesses demonstrated roles in promoting neuronal differentiation, neurite outgrowth, and cell viability [27]. Functionally, CNTF regulates oxidative stress, inflammation, and other cellular processes to support regeneration and provide protective effects in the central nervous system (CNS) [28]. Mechanistically, CNTF first binds to its specific receptor α subunit (CNTFRα), which then recruits glycoprotein 130 (gp130) and leukemia inhibitory factor receptor β (LIFRβ) to assemble a heterotrimeric receptor complex [29]. CNTF binding to its receptors mainly activates the Janus kinase (JAK)/signal transducer and activator of transcription (STAT) signaling pathway, along with additional cascades such as the phosphoinositide 3-kinase (PI3K)/serine/threonine-specific protein kinase (Akt) or mitogen-activated extracellular signal-regulated kinase (MEK)/extracellular regulated protein kinase (ERK) pathways, mitogen-activated protein kinases (MAPK), AMP-activated protein kinase (AMPK), depending on the cell type [30,31]. For instance, PI3K/Akt pathway triggered by CNTF has a crucial impact in both neuronal and neurite growth, and mediates the CNTF’s role in glucose uptake within skeletal muscle tissue [32].

MANF was initially discovered as an ER-localized protein with protective functions for dopamine neurons, and CDNF is a structural homolog of MANF, both of which constitute a novel family of evolutionarily conserved neurotrophic factors [24,33]. Despite their neurotrophic properties, MANF and CDNF do not share any sequence similarities with traditional NTFs. They exhibit dual functions: acting both as an extracellular trophic factor and as an ER-resident protein [34]. Neuroplastin (NPTN) has been identified as a cell surface receptor for MANF, mediating its effects [35]. Binding of MANF to NPTN regulates ER stress-mediated cell death and inflammatory response through the activation of the NF-κB signaling pathway [35]. However, CDNF has not yet been found to bind to any cell surface receptors. Notably, proper protein folding within the myocardial ER is essential for maintaining cardiac function. Various pathological conditions, such as cardiac hypertrophy, ischemia, heart failure (HF) that induce ER stress, triggers the accumulation of unfolded or misfolded protein, which ultimately leads to the unfolded protein response (UPR) [36]. The UPR originally plays a pro-survival role, whereas under prolonged stress conditions, sustained UPR activation can switch to promote cell death [37]. The present studies have demonstrated that MANF and CDNF can modulate ER stress-induced UPR and supports protein homeostasis [38,39]. However, the potential mechanisms by which MANF and CDNF reduce the activation of UPR remain unclear, although there are some relevant researchers suggesting a connection to glucose regulatory protein 78 (GRP78) [40]. MANF interacts with GRP78 in the ER and reduced ER calcium levels facilitates the secretion of MANF. MANF then binds to the nucleotide binding domain of GRP78 [41,42]. MANF also inhibits ADP dissociation from GRP78 and impedes ATP binding. The activity stabilizes GRP78-client protein complexes, a step to promote their transfer to downstream [43]. Consequently, depletion of MANF is predicted to destabilize these complexes, rendering cells more prone to activating the UPR. Conversely, MANF overexpression likely reinforces complex stability and attenuates UPR induction. Nevertheless, one study has challenged this paradigm, asserting that MANF and GRP78 interact directly regardless of calcium levels [44]. Therefore, further investigations into MANF and the related protein CDNF are required to elucidate their precise roles as negative regulators of the UPR across diverse ER stress contexts.

## 3. The Roles of Neurotrophic Factors in Cardiovascular Pathology

**Figure 1:**
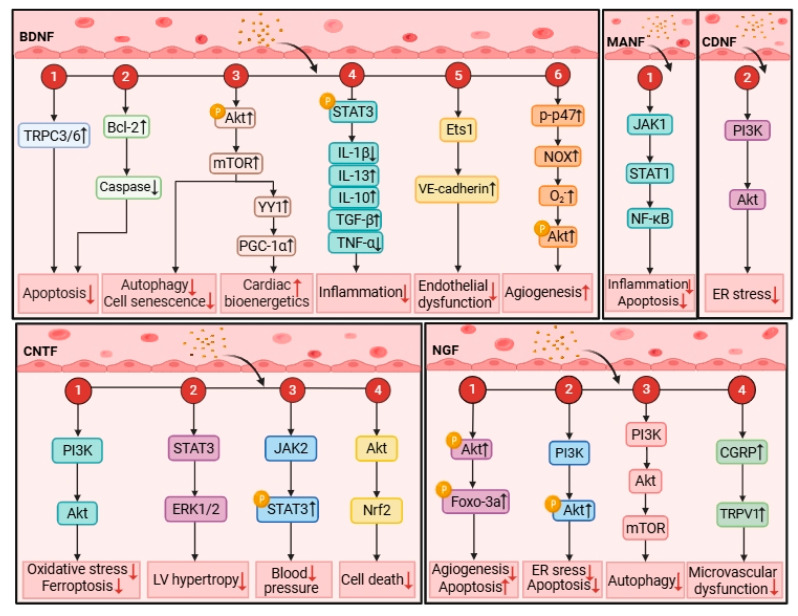
**The roles of NTFs in CVDs**. NTFs have been demonstrated to play a pivotal role in the regulation of cardiac remodeling and cardiac function, achieved through abundant pathophysiologic processes, including angiogenesis, oxidative stress, inflammation, ER stress, apoptosis, mitochondrial dysfunction, autophagy, and cell death. This figure was created in BioRender. Akt, serine/threonine-specific protein kinase; BDNF, brain-derived neurotrophic factor; CNTF, ciliary neurotrophic factor; CDNF, cerebral dopamine neurotrophic factor; CGRP, calcitonin gene-related peptide; Caspase, cysteinyl aspartate specific proteinase; ERK, Extracellular regulated protein kinase; Foxo, forkhead box-O transcription factors; ER, endoplasmic reticulum; IL-1β, interleukin-1β; IL-13, interleukin-13; IL-10, interleukin-10; JAK, Janus kinase; LV, left ventricle; MANF, mesencephalic astrocyte-derived neurotrophic factor; mTOR, mammalian target of rapamycin; NT-3, neurotrophin-3; NF-κB, nuclear factor kappa-B; NGF, nerve growth factor; NOX, NADPH-oxidase; PI3K, phosphatidylinositol 3-kinase; TRPV1, transient receptor potential vanilloid-1; STAT, signal transducer and activator of transcription; TRPC, transient receptor potential canonical; TGF-β, transforming growth factor-β; TNF-α, tumor necrosis factor-α; VE-cadherin, vascular endothelial cadherin.

NTFs are weakly expressed in healthy human hearts, while their expressions significantly change in cardiomyocytes and cardiac tissues responding to cardiovascular injury (Table 1). The exact cell types contributing to the change are not known, but might be endothelial cells, smooth muscle cells, platelets, macrophages and cardiomyocytes [45,46,47]. The alterations of circulating and cardiac NTF concentrations are established biomarkers for atherosclerosis, hypoxic–ischemic damage, heart failure, cardiac hypertrophy and endothelial dysfunction [48]. As has been demonstrated extensively, NTFs are biomarkers associated with unfavorable prognosis of CVDs. Thus, subsequent sections will examine NTFs’ potential roles regarding CVDs (Figure 1).

### 3.1. Coronary Artery Disease

BDNF reflects the lipid imbalance, inflammatory state and stenosis degree in patients with coronary artery disease (CAD), and it is negatively correlated with the levels of representative molecules [49]. The upregulation of BDNF, with elevated levels of negative signaling regulators and diminished TrkB expression in perivascular fat, indicates a compromised or completely absent vascular BDNF signaling pathway in patients with coronary atherosclerosis [50]. Of note, the heightened expression of BDNF serves to mitigate the atherosclerosis in diabetes mellitus patients by promoting M2 macrophage polarization through the activation of the CAMP response element binding protein/BDNF/TrkB signaling cascade while simultaneously suppressing STAT3 pathway [46]. Additionally, BDNF binding to TrkB plays a crucial role in preserving endothelial integrity throughout the atherosclerotic process by boosting Ets1-driven vascular endothelial cadherin (VE-cadherin) expression and enhancing smooth muscle cell function, allowing both mechanisms to act together in the development of CAD [51]. Plasma BDNF levels were enhanced, and BDNF expression was elevated in coronary arteries in patients with unstable angina (UA) [52]. Paradoxically, plasma BDNF contents are significantly lower in patients with CAD or in those with stable angina (SA) and acute myocardial infarction (AMI), and reduce along with clinical acuity and disease severity [53]. However, the change in plasma BDNF levels only in CAD patients or in those with SA and AMI remains greatly controversial. On the one hand, BDNF was demonstrated to be increased within the initial hours after AMI, and positively correlated with the severity of subsequent HF [54]. On the other hand, decreased BDNF concentrations were linked to adverse cardiac remodeling after AMI [55]. According to these findings, either a protective or deleterious effect of BDNF on CAD has been proposed. Nevertheless, all these studies classified BDNF concentrations just based on the clinical manifestations in CAD patients, disregarding the morphological characteristics of their coronary plaques. Therefore, differences in plaque features may account for the conflicting BDNF patterns observed in previous studies [56,57]. Interestingly, elevated plasma BDNF levels are independently associated with a vulnerable plaque phenotype, irrespective of clinical CAD presentations [48]. The phenomenon is demonstrated to be related to increased oxidative stress by BDNF [52,53]. In addition, inflammatory responses, platelets activation and endothelial dysfunction particularly in patients with CAD can also affect or be affected by BDNF [57,58]. Thus, all these elements could act as modulators that must be taken into account when assessing BDNF contents and its association with plaque vulnerability [59,60]. However, further investigations are needed to deeply illustrate the role of BDNF in coronary plaque activity and stability.

Although there are a few studies describing the effects of NGF, it has been implicated in the development of CAD. The elevated levels of NGF levels positively correlate with disease activity, inflammatory state, and plaque instability [61]. By binding to its TrkA and p75^NTR^, NGF acts as a dual driver. On one hand, it facilitates smooth muscle cells’ migration and proliferation, and impairs endothelial function, which contributes to early plaque formation and later vascular remodeling [62]. On the other hand, it activates mast cells and macrophages to exacerbate inflammatory responses, accelerating plaque progression [63]. Briefly, NGF serves as a negative regulatory molecule when modulating the CAD’s progression.

### 3.2. Myocardial Infarction

The BDNF-TrkB axis suppresses myocardial apoptosis and mitigates cardiac ischemic injury through modulating transient receptor potential canonical 3/6 (TRPC3/6) channel [54]. BDNF also upregulates b-cell lymphoma-2 (Bcl-2) expression and downregulates cysteinyl aspartate specific proteinase 3 (caspase-3) activity to suppress apoptosis [54]. However, the upregulation of miR-195 in ischemic cardiomyocytes can facilitate pro-apoptotic effects via targeting Bcl-2 [64]. Notably, dysregulation of the brain–heart axis has been increasingly implicated in the onset and progression of myocardial infarction (MI) [65,66,67]. Cardiac afferent nerve fibers transmit the ischemic signals of MI to the brain, leading to the activation of various signaling pathways in the CNS. Activation of these CNS pathways enhances BDNF expression within the brain, then increases in circulating BDNF in turn confers protective effects on the heart during ischemic injury [68]. Increases in BDNF contribute to improved survival and attenuated left ventricular (LV) remodeling, an effect mediated through the reduction in early inflammatory response and pathological angiogenesis. Furthermore, the BDNF Val66Met gene polymorphism leads to adverse cardiac remodeling after MI and causes pro-inflammatory M1-like phenotypes in both mouse and human macrophages [55]. Simultaneously, it is also associated with poorer functional recovery, including greater susceptibility to depression and anxiety, impaired cardiac autonomic modulation, and attenuated improvement in left ventricular ejection fraction, indicating BDNF polymorphism are closely connected with the prognosis of CAD [69,70]. BDNF exerts pro-angiogenic effects through activating Akt, thereby promoting the generation of reactive oxygen species (ROS) derived from nicotinamide adenine dinucleotide phosphate hydrogen (NADPH) oxidase, and subsequently facilitating angiogenic tube formation [58]. Of note, BDNF and vascular endothelial growth factor (VEGF) can cooperate to facilitate tube formation [71]. Moreover, short-term BDNF stimulation promotes angiogenesis and tube formation by inducing moderate ROS production. In contrast, prolonged exposure reduces cell viability due to the excessive ROS. Beyond activating Akt in vascular endothelial cells through the TrkB-dependent mechanism, BDNF also promotes neovascularization through the recruitment of bone marrow-derived cells [72,73]. Development of cardiac sympathetic heterogeneity after MI contributes to ventricular arrhythmias and sudden cardiac death. BDNF plays protective roles by preferentially binding p75^NTR^ to stimulate sympathetic denervation in the peri-infarct region and inhibits hyperinnervation in the distal peri-infarct area [74]. The downregulation of miR-18a elevates BDNF expression, thereby inhibiting the Akt/mammalian target of rapamycin (mTOR) signal pathway [75]. Consequently, this induction of autophagy and suppression of cell senescence confers protection against acute MI [75].

NGF with its high-affinity receptor TrkA activates the Akt/Forkhead box-O transcription factors (Foxo-3a) signaling pathway, thereby promoting angiogenesis and suppressing apoptosis [73,76]. Following MI, the infarct site exhibits a significant elevation in NGF levels accompanied by the upregulation of growth-associated protein 43 (GAP43), a key driver of post-MI cardiac nerve sprouting [77]. NGF and GAP43 are retrogradely transported to the left stellate ganglion, thereby inducing sympathetic hyperinnervation [77,78]. Individual variability in NGF expression may be partly responsible for differences in nerve sprouting and arrhythmia susceptibility after MI. However, empagliflozin can mitigate these adverse effects by inhibiting the NGF/TrkA pathway [79]. Of note, proNGF binds p75^NTR^ to promote pathological changes in the microvasculature that ultimately result in cardiomyopathy after fatal MI [80].

CNTF can markedly reduce oxidative stress, ferroptosis, and cardiac dysfunction in MI-induced cardiac remodeling via the PI3K/Akt signaling in murine models [81]. Cardiac glucose metabolism in leptin-resistant mouse hearts can be restored after acute MI via CNTF-mediated STAT3/PI3K/Akt signal pathway [82]. CNTF also reverses LV hypertrophy in leptin-deficient and leptin-resistant obese mice, involving a reduction in ventricular septal thickness, posterior wall thickness, and LV mass [82]. Furthermore, both CNTF and leptin have been demonstrated to activate the STAT3 and ERK1/2 signaling pathways [83].

Collectively, these studies show that BDNF, NGF and CNTF serve a protective role in MI through specific cellular-protein-receptor interactions.

### 3.3. Heart Failure

Emerging clinical evidence indicates an inverse relationship between circulating BDNF levels and lethality in patients with HF, wherein diminished BDNF concentrations are associated with poor survival prognosis and cardiac remodeling [84]. Consistently, a recent systematic review and meta-analysis specifically evaluated circulating BDNF levels in HF patients compared with healthy individuals, and discussed its potential as a diagnostic, stratification, or prognostic biomarker, demonstrating that BDNF is significantly lower in HF patients [85]. Notably, BDNF levels reduce in HF, while an increase in acute HF may be associated with inflammatory response. BDNF loss triggers chronic ischemic HF and TrkB agonists can counteract ischemic LV dysfunction via replenishing myocardial BDNF. β3 adrenergic receptor stimulation increases cardiac BDNF levels and plays a crucial role in post-ischemic cardiac BDNF production [86]. BDNF binding to TrkB initiates a calcium/calmodulin-dependent protein kinase II (CaMKII)-dependent signaling cascade, acting with β-adrenergic signaling to enhance myocardial Ca^2+^ cycling, thereby augmenting cardiac contraction and relaxation [87]. Interestingly, a parallel study revealed that the modulation of cardiac contractility by BDNF is facilitated through the truncated TrkB. T1 receptors present on cardiomyocytes, a discrepancy likely explained by differences in cardiac phenotypes between the mouse models used in these studies [88]. One study has demonstrated that BDNF upregulates peroxisome proliferator-activated receptor γ coactivator 1α (PGC-1α) expression and recovers cardiac bioenergetics through activating AKT/mTOR/Yin Yang 1 (YY1) pathway in response to HF, whereas exercise can work synergistically with BDNF [89]. Moreover, the downregulation of silent information regulator 1 in cardiomyocytes activates nuclear factor kappa-B (NF-κB), which upregulates miR-155 [90]. Elevated miR-155 expression suppresses BDNF production, thereby contributing to impaired ventricular function in HF [91].

NGF promotes cardiac regeneration by stimulating cardiomyocyte proliferation rather than possessing anti-apoptotic effect, thereby reducing the incidence of HF and mortality [92]. Studies indicate that abnormal cardiac sympathetic nerves are observed in chronic HF, along with reduced expression of NGF and NT-3, whereas there is increased expression of BDNF and CNTF [93]. Differential expression of cardiac NTFs may explain two features of sympathetic dysfunction in chronic HF: sympathetic loss and nerve terminal transformation [94]. The absence of NGF and NT-3 may serve as the initial trigger for altered cardiac sympathetic function, while the upregulation of BDNF and CNTF may promote the shift in sympathetic nerves from a balanced norepinephrine (NE) storage/release/uptake mechanism to a predominantly NE-releasing mechanism [95].

### 3.4. Ischemia/Reperfusion Injury

Ischemia/reperfusion (I/R) injury can dysregulate sarcoplasmic reticulum in the heart and calcium of the ER, thus leading to MANF secretion [96]. The present study hypothesizes that elevated MANF alleviates ER stress through the inhibiting JAK 1/STAT1/NF-κB signal pathway to mitigate cardiomyocyte apoptosis [97]. The JAK1/STAT1 signaling pathway can regulate inflammation and LV remodeling subsequent to MI, ultimately mitigating I/R injury in cardiac tissues [98]. Similarly to MANF, CDNF binding to KDEL activates the PI3K/Akt signal pathway following myocardial I/R to mitigate ER stress, restore the calcium transient, and reduce the infarct area [99]. However, the lack of N-glycosylation on the CDNF protein reduces the protein stability and promotes cardiomyocyte apoptosis through downregulating the PI3K/Akt pathway [100]. In addition, BDNF and NT-3 have been implicated in protection against ischemia and I/R-induced cardiomyocyte death. One in vivo study demonstrates that the adrenoceptor beta 2 agonist and caveolin-3 can potentiate the activity of the BDNF/TrkB and cyclic adenosine 2′,3′-monophosphate/protein kinase signaling pathways in the diabetic heart [101]. Meanwhile, another study has revealed that NT-3 reduces apoptosis via the ERK-Bim signaling pathway and promotes angiogenesis [102]. Consistent with these findings, NGF binding to TrkA protects against I/R injury by alleviating ER stress-induced cardiomyocyte apoptosis through the activation of the PI3K/Akt signaling pathway [103]. NGF also inhibits autophagy during myocardial I/R by the activation of its downstream PI3K/Akt/mTOR signaling. Simultaneously, NGF can enhance cardiac recovery by upregulating transient receptor potential vanilloid-1 (TRPV1) and promoting the synthesis and release of endogenous calcitonin gene-related peptide (CGRP) [104,105]. As mitochondrial homeostasis is disrupted, CNTF prevents myocardial cells against oxygen-glucose deprivation followed by re-oxygenation by Akt-Nuclear factor erythroid 2-related factor 2 signaling pathway to avoid ischemic injury [106,107].

### 3.5. Cardiac Hypertrophy

Hypertension is a prevalent cause of cardiac hypertrophy, predominantly affecting left ventricular hypertrophy [108]. Elevated circulating BDNF concentrations are associated with an increased risk of hypertension and cardiometabolic dysfunction in middle-aged and elderly adults, while endothelial BDNF expression is found to decrease [109]. These findings imply that the elevated serum/plasma BDNF observed in hypertension is potentially not from the endothelial cells [108]. BDNF can intervene cardiovascular stress response, affect heart rate and regulate blood pressure in which effects on blood pressure fluctuations could be achieved through renin-angiotensin signal transduction pathway. In addition, CNTF also modulates blood pressure by regulating angiotensin II (Ang II)-induced pressure response through a JAK2/STAT3-dependent mechanism, suggesting that CNTF can effectively prevent the occurrence of hypertension [110]. Of note, cardiac hypertrophy promotes the expression of MANF, then MANF protects the heart through maintaining the glycolysis-oxidative phosphorylation balance and mitochondrial homeostasis by interacting with the pro-apoptotic protein bcl2-associated X (BAX) to inhibit mitochondrial translocation, thereby reducing mitochondrial damage and cardiomyocyte death [111]. Meanwhile, MANF effectively counteracts the hypertrophic effects triggered by α1-adrenergic receptor agonist phenylephrine. While the precise mechanisms behind MANF’s anti-hypertrophic actions remains to be elucidated, MANF demonstrates protective capabilities when added to cultured cells, at least some of its benefits mediated through a cell-surface receptor (e.g., NPTN) [35,40].

## 4. Therapeutic Approaches

Having distinct mechanisms of action, NTFs facilitate cardiomyocyte repair, safeguard cardiac tissue, and bolster cellular function. Additionally, they trigger receptor activation that initiates cascades of reactions following cardiac damage, contributing to enhanced cardiac functions and improved cardiac remodeling. Given all these characteristics of NTFs, the following section will focus on the exploration of NTFs with therapeutic effects that can be applied in CVDs (Table 2).

### 4.1. Exercise

Exercise has emerged as a prevalent approach for the prevention and management of chronic diseases [117,118,119]. A large body of research has supported that exercise exerts considerable effects on NTFs. Exercise mitigates the decline in ejection fraction (EF) and the elevation in LV end-diastolic pressure (LVEDP) and increases stroke volume and cardiac index following MI [113]. The improvement of EF but not LVEDP is mediated through the activation of the BDNF-TrkB signal pathway and its downstream effectors Ca^2+^/CaMKII and Akt in the noninfarct LV area [113]. High-intensity interval training (HIIT), resistance training (RT), and combined exercise elicit more pronounced alterations in NT levels relative to baseline measurements [120,121]. Interestingly, both the combined training and RT regimens induce greater modifications in BDNF, NT-3, and NT-4/5 compared to the HIIT group [122]. Consistently, both continuous training regimens and HIIT elevate circulating levels of BDNF and GDNF, and the increases are consistently greater after HIIT protocols compared to continuous training regimens [123]. Moreover, serum BDNF concentrations have been observed to increase in response to acute and chronic aerobic exercise, indicating that aerobic but not strength/resistance training generally increases peripheral BDNF concentrations [124,125]. Long-term aerobic exercise also can increase NT-4/5 expression. Notably, according to studies, increased serum BDNF induced by acute exercise may be connected with the increased number of platelets entering the bloodstream after their release from the spleen, and plasma proBDNF in response to high-intensity exercise is released from skeletal muscle [47,125]. In fact, exercise triggers the circulating increase in BDNF levels through several tissue sources including lungs, intestinal tissue, myocardial cells, endothelial cells, skeletal muscle, peripheral neurons, platelets, and the brain [124]. Nevertheless, evidence about the effect of exercise on circulating BDNF levels is limited and controversial, and more investigations are urgently needed to bridge the research gap.

**Figure 2:**
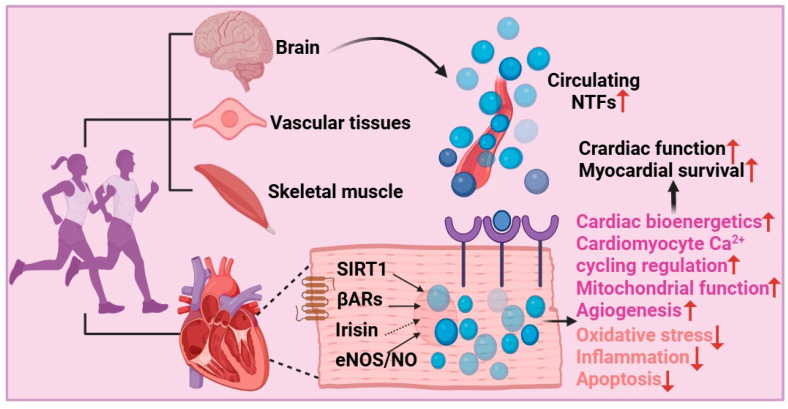
**Exercise activates the regulation of NTFs for cardioprotection**. Exercise modulates the secretion of NTFs to confer cardioprotective effects via organ communication, which eventually improves cardiac function and increases myocardial survival by boosting mitochondrial function, regulating cardiomyocyte Ca^2+^ cycling, promoting angiogenesis, maintaining cardiac bioenergetics and attenuating oxidative stress, inflammation, and apoptosis. This figure was created in BioRender. eNOS, endothelial nitric oxide synthase; HO-1, heme oxygenase 1; NO, nitric oxide; SIRT1, silent mating type information regulation 2 homolog-1; βARs, β-adrenergic receptors; →, invovled; ⇢, uncertain.

Therefore, when formulating exercise programs for patients with heart disorders, NTFs could be used as biomarkers and various characteristics must be taken into consideration [126,127] (Figure 2). However, most studies emphasize the relationship between exercise and NTs, leaving other NTFs largely unexplored. Expanding this scope to encompass MANF and CDNF could provide novel insights into exercise-induced secretion of NTFs. In response to adverse effects, such as ER stress, the expression of MANF and CDNF is upregulated, which in turn can alleviate ER stress. In parallel, CDNF also reduces protein aggregation and attenuates inflammation. Although there are no relevant studies, future investigations can focus on how different exercise intensities influence the circulating levels of MANF and CDNF and what functions the alterations can exert.

### 4.2. Small Molecule Agonist/Mimetic

**Figure 3:**
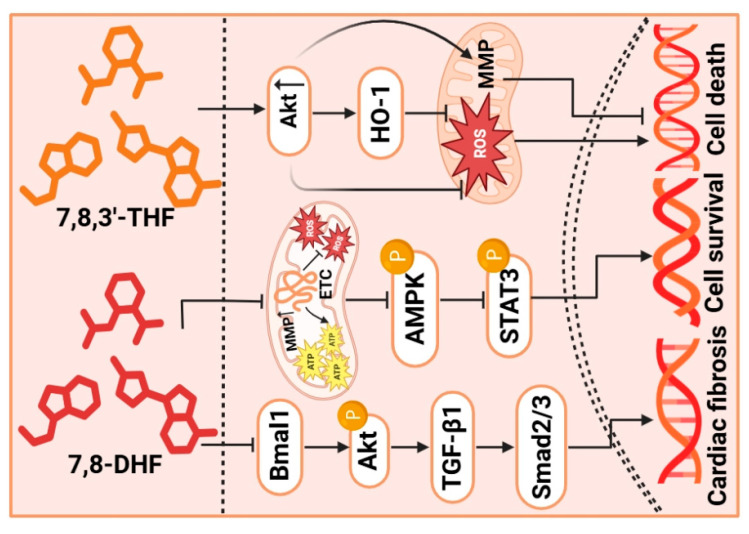
**The roles of small molecule agonists/mimetics in modulating cardiac function**. This illustrates that 7,8-DHF and 7,8,3′-THF activate key signaling pathways, including Akt, AMPK, and STAT3 to promote cell survival. Concurrently, they can also inhibit the TGF-β1/Smad2/3 axis, thereby attenuating cardiac fibrosis. This figure was created in BioRender. AMPK, AMP-activated protein kinase; Akt, serine/threonine-specific protein kinase; Bmal1, brain and muscle Arnt-like protein 1; MMP, mitochondrial membrane potential; STAT, signal transducer and activator of transcription; TGF-β1, transforming growth factor-β1; 7,8,3′-THF, 7,8,3′-Trihydroxyflavone; 7,8-DHF, 7,8-Dihydroxyflavone.

TrkB receptor agonist can enhance ischemic LV dysfunction by restoring myocardial BDNF content. Thus, targeting the TrkB receptor agonist has emerged as a promising therapeutic strategy to restore BDNF-dependent neurotrophic support to the ischemic myocardium [128]. 7,8-Dihydroflavone (7,8-DHF) acts as both a TrkB receptor agonist and mimic of BDNF [129,130]. Emerging evidence indicates that 7,8-DHF effectively reduces cardiac fibrosis as well as maintains healthy circadian rhythms, partly stemming from its ability to suppress the brain and muscle Arnt-like protein 1 (Bmal1)/Akt signaling pathway [131]. Furthermore, 7,8-DHF has been shown to have mito-protective effects on rotenone (Rot)-induced cardiomyocyte toxicity. These effects are a result of the inhibition of mitochondrial dysfunction and the promotion of the nuclear translocation of phosphorylated STAT3 [112]. Similarly, 7,8-DHF also can enhance cardiac function and restore the expression of related proteins by activating the Akt signaling pathway against doxorubicin (Dox)-induced injury and cell death [114]. Of note, 7,8-DHF is orally bioactive and processes therapeutic efficacy when BDNF signaling is deficient [130]. In addition, 7,8,3′-Trihydroxyflavone (7,8,3′-THF) is another BDNF mimic. Both 7,8-DHF and 7,8,3′-THF have the ability to facilitate the recovery of palmitic acid (PA)-induced mitochondrial damage by maintaining the function of Akt-dependent mitochondria [115]. In conclusion, these studies demonstrate that TrkB receptor agonists ameliorate various cardiac pathologies primarily through the restoration of mitochondrial function and activation of the Akt signaling pathway (Figure 3).

However, the translation of 7,8-DHF to clinical application has a series of critical hurdles. First, due to its poor pharmacokinetics, characterized by low oral bioavailability and rapid metabolism, this behavior hinders the maintenance of therapeutic concentrations at lesion sites [130]. Furthermore, it is important that the dosage of 7,8-DHF must be validated strictly as over-activation of the TrkB signaling may pose devastating consequences, like off-target risks [130]. At the same time, long-term TrkB activation also leads to unknown risks, such as tumorigenesis and cardiovascular hyperplasia [132]. Therefore, the progression from bench to bedside will depend on structural optimization of the compound, the development of precise delivery systems, and in-depth mechanistic studies.

### 4.3. Gene Therapy

Harnessing viral or non-viral vectors for gene transfer has been the mainstay of medical therapy. NTFs gene transfer leads to functional improvement and normal development with the heart [133]. A particular investigation compared the impact of elevated cardiac NGF levels using two distinct methodologies: direct intramyocardial administration of an adeno associated virus (AAV) 2 vector and whole-body AAV9 delivery [134]. The results demonstrated that NGF gene transfer successfully shielded cardiac tissue from damage associated with diabetic cardiomyopathy (DCM), regardless of the method employed [134]. However, the overexpression of CNTF in cardiomyocytes mediated by AAV9 exacerbates apoptosis, cardiac fibrosis, and inflammatory response, suggesting that CNTF gene delivery may fail to improve adverse cardiac remodeling in diabetic mice [116]. Additionally, intrathecal lentivirus-mediated interference RNA targeted at reducing NGF gene expression attenuates myocardial I/R injury in the rat model, which is achieved through the inhibition of TRPV1/Akt/ERK signal pathway [104].

### 4.4. Stem Cell Therapy

**Figure 4:**
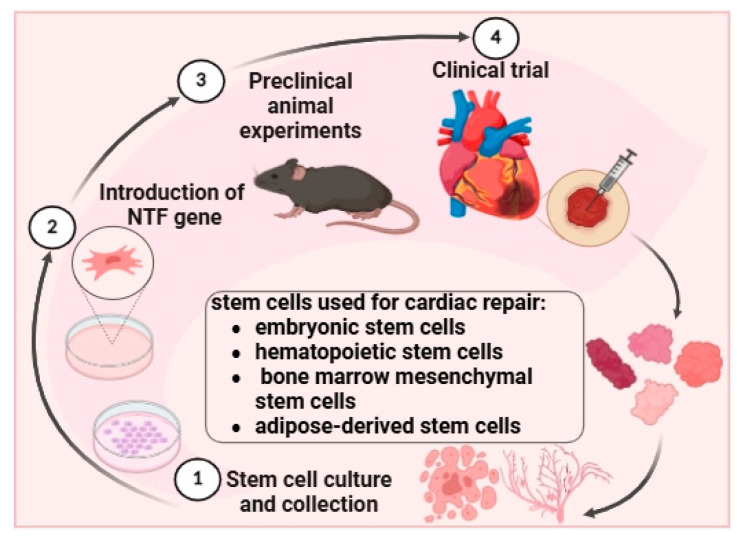
**The overview of NTF-modified stem cell therapeutic strategy for cardiac repair**. Several types of stem cells, including embryonic stem cells, hematopoietic stem cells, bone marrow mesenchymal stem cells, and adipose-derived stem cells, are considered as potential candidates for myocardial regeneration. One pivotal step is to introduce NTF genes into cells, which then undergo preclinical trials in animal models before clinical trials to evaluate safety and effectiveness in human patients. This figure was created in BioRender.

Autologous and allogeneic cell transplantation has been applied in cardiac generation, substantially mitigating concerns related to immune rejection [135]. Current stem cell types for cardiac repair are embryonic stem cells, hematopoietic stem cells, bone marrow-derived mesenchymal stem cells, and adipose-derived stem cells [136] (Figure 4). Notably, the exposure to NTF can rejuvenate aged human multipotent mesenchymal stromal cells (hMSCs) [137]. Transplantation of these NTF-rejuvenated hMSCs improves cardiac function, primarily by suppressing apoptosis and stimulating angiogenic responses [138]. The expression of NGF significantly increases after mesenchymal stem cells transplantation, which will affect sympathetic remodeling and the electrophysiological properties after MI [137]. NTF-rejuvenated stem cells can serve as an effectively dual product for integrating cellular and genetic treatments to enhance aged heart recovery following cardiac ischemia. However, long-term observation is needed to investigate potential side effects and efficacy.

### 4.5. Recombinant Protein Therapy

The administration of recombined BDNF can elevate myocardial BDNF levels, thereby enhancing survival rate and improving cardiac function, with a concomitant reduction in oxidative stress and myocardial apoptosis [139]. Recombined BDNF also effectively ameliorates the compromised exercise capacity observed in mice with MI-induced HF, through enhancing fatty acid oxidation by activating theAMPKα-peroxisome proliferator-activated receptor gamma coactivator 1-alpha signaling pathway [140]. The underlying mechanism resembles that of exercise training, thus BDNF is considered a potential therapeutic target for enhancing exercise capacity following HF. Consequently, therapeutic intervention with BDNF, whether as a monotherapy or in conjunction with exercise training, is a promising strategy in this context [141]. Furthermore, recombinant MANF treatment also decreases infarct size and inflammation in I/R injury. The knockdown of endogenous MANF will increase cell death, but adding recombinant MANF restores the protective effect. However, the clinical application of recombinant NTFs is constrained by certain limitations [139,142]. Currently, the primary challenge includes the short half-life, poor targeting, poor stability, expensive cost and delivery barriers. However, the production of recombinant NTFs is of great biotechnological interest. Notably, one study has demonstrated that recombinant BDNF can be efficiently produced through the engineering of Escherichia coli, representing an approach with high yield and low cost to producing and purifying recombinant NTFs [45]. In addition, there are other new strategies that are widely concerned, including nanotechnology, protein engineering, and fusion technology [143,144,145]. Although these methods can achieve the goal of producing a stable and biologically active protein, more researchers are still needed for pharmaceutical application.

## 5. Conclusions

NTFs have garnered attention for elucidating connections between CVD pathogenesis and therapeutic approaches, while offering broad applications in disease management. Among these, BDNF and MANF seem to hold the greatest translational promise, reflecting their roles in cardioprotection. Current studies indicate that NTFs essentially safeguard cardiomyocytes in the heart through three key mechanisms. First, NTFs directly participate in mediating cardiac normal contractility and relaxation. Second, NTFs mitigate the detrimental impacts of fibrosis, hypertrophy, apoptosis, cell death, oxidative stress and inflammation, effectively preserving cardiomyocytes. Finally, NTFs promote angiogenesis and maintain endothelial function. All three factors foster neuro-immune–cardiovascular interaction under cardiac stress, which are intricately related to CVD development. Meanwhile, in contrast to pathological conditions, studies have demonstrated that some certain NTFs, such as BDNF, NT-3, NT4/5, and GDNF, can be stimulated to increase secretion through exercise. Additionally, multiple transcription factors are involved in the regulation of ER stress-related diseases. Thus, elucidating the targets of MANF/CDNF in the context of ER stress in the heart represents a significant step into therapeutic strategies, particularly their interactions with activating transcription factor 6.

With respect to clinical translation, due to NTFs being able to be produced and isolated using external plasmids, it is feasible to manufacture recombinant NTFs on an industrial scale, even gene-engineered cells. Moreover, exploring the development of small molecular agonist/mimetics is feasible. Notably, beyond the use of a single NTF, further investigations can focus on the benefits of multiple factor synergistic approaches, especially in stem cell therapies. In these ways, people can be supplemented with some deficient NTFs in a specialized manner.

Looking ahead, several research aspects should be taken into account. To begin with, finding proper methods of differentiating biological differences between the precursor/mature form and detecting each NTF concentration will be critical to ensure accurate interpretation of therapeutic efficacy. Moreover, due to the different NTFs’ properties, integrative approaches hold promise for synergistically enhancing NTF signaling and improving intervention results. For instance, exercise combined with specific NTF supplements or stem cell therapy combined with gene therapy can aid more efficient treatment outcomes. In addition, optimizing the design of emerging NTF vectors is required for evaluating durability, immune responses, and assessing long-term safety. Furthermore, it is important to identify better predictive biomarkers to stratify patient subgroups, enabling them to most likely benefit from NTF-targeted interventions.

However, the understanding of the roles of NTFs in CVDs remains limited, with the precise molecular pathways still shrouded and not already completely observed. Therefore, the application of NTF products in CVDs needs more research to identify additional targets and optimize delivery approaches, providing standards for clinical applications.

## Figures and Tables

**Table 1:** Summary of the changes in circulating NTFs levels in patients with CVDs.

	CAD	MI	AHF	CHF	I/R Injury	CH
**BDNF**	?	↑	↑	↓	↑	↑↓
**NGF**	↑	↑	↑	↓	↑	↑
**NT-3**	→	↑	→	↑	→	↑
**NT-4/5**	→	↑	→	→	→	→
**GDNF**	↑	↑	↑	→	↑	↑
**CNTF**	→	↑	→	↑	→	↑
**MANF**	↑	↑	→	↑	↑	↑
**CDNF**	→	↑	→	→	↑	→

Note: ↑: increased; ↓: decreased; ?: controversial; →: uncertain; CAD: coronary artery disease; MI: myocardial Infarction; AHF: acute heart failure; CHF: chronic heart failure; I/R: ischemia/reperfusion; CH: cardiac hypertrophy.

**Table 2:** The applications of NTFs in therapy for different CVDs.

Neurotrophic Factors	Cardiovascular Diseases	Stage of Evidence	Applications	Main Results	Refs.
BDNF	Rot-induced cytotoxicity in cardiomyocytes	In vivo	7,8-DHF regulates AMPK activity and promotes p-STAT3 nuclear translocation	Preserving mitochondrial function and attenuating oxidative stress	[112]
	MI	In vitro and vivo	Exercise promotes angiogenesis and confers cardioprotective effects by activating the BDNF/TrkB signaling axis in an eNOS/NO-dependent manner	Increasing myocardial angiogenesis and improving cardiac function	[113]
	DMAS	In vitro	BDNF overexpression drives M2 macrophage polarization and mitigates DMAS progression through STAT3 pathway inhibition.	Reducing the atherosclerosis lesion area and alleviating inflammatory response	[46]
	MI	In vitro and vivo	BDNF/TrkB alleviates cardiac ischemic injury and inhibits cardiomyocytes apoptosis by regulating TRPC3/6 channels	Reducing infarct size and improving cardiac function	[54]
	Dox-induced cardiotoxicity	In vivo	7,8-DHF attenuates Dox-induced cardiotoxicity by activating Akt/STAT3/AMPK/ERK signals, and increasing mitochondrial oxidative phosphorylation	Improving cell vitality and reducing cell death	[114]
	PA-induced injury in cardiomyocytes	In vivo	7,8-DHF and 7,8-THF attenuate PA-induced injury in cardiomyocytes by alleviating mitochondrial impairments through activating Akt signaling	Restoring cell viability and improving mitochondrial function	[115]
NGF	MI	In vitro and vivo	The prosurvival Akt/Foxo pathway mediates the therapeutic effects of NGF, whose overexpression upregulates stem cell factor expression	Enhancing endothelial and cardiomyocyte survival, promoting neovascularization, and improving myocardial perfusion and cardiac function	[76]
NT-3	I/R injury	In vivo	NT-3 inhibits I/R-induced cardiomyocyte apoptosis through increasing the ERK and reducing the Bim level	Inhibiting cardiomyocyte apoptosis, and promoting angiogenesis	[102]
CNTF	MI	In vivo	CNTF controls MI-induced cardiac remodeling via initiating the PI3K/Akt signaling pathway	Preventing cardiac hypertrophy and cardiac fibrosis, and reducing oxidative stress and ferroptosis	[81]
	DCM	In vivo	AAV9-based cardiac CNTF gene delivery can adverse ventricular remodeling in diabetic mice models	Inducing CNTF gene expression, promoting inflammation, increasing interstitial and perivascular areas, and exacerbating cell apoptosis and cardiac fibrosis	[116]
MANF	I/R injury	In vitro and vivo	MANF alleviates myocardial I/R injury by inhibiting ER stress and the JAK1/STAT1/NF-κB pathway	Reducing inflammation, maintaining cellular function, and reducing ER stress-induced cell death	[76]
	Cardiac hypertrophy	In vitro and vivo	MANF interacts with BAX to inhibit mitochondrial translocation and regulates the balance between glycolysis and oxidative phosphorylation, maintaining mitochondrial homeostasis	Reducing mitochondrial damage and apoptosis	[111]
CDNF	I/R injury	In vitro and vivo	CDNF induces cardioprotection via KDEL receptor binding and PI3K/Akt activation	Reducing ER stress, restoring the calcium transient, and avoiding mitochondrial impairment, and reducing the infarct area	[99]

**Summary of cardiovascular outcomes treated with NTFs**. This includes the variety of NTFs, typical animal models, relevant mechanisms, and main study findings. AMPK, AMP-activated protein kinase; AAV, adeno associated virus; Akt, serine/threonine-specific protein kinase; BDNF, brain-derived neurotrophic factor; BAX, bcl2-associated X; CDNF, cerebral dopamine neurotrophic factor; CGRP, calcitonin gene-related peptide; CNTF, ciliary neurotrophic factor; DMAS, diabetes mellitus-accelerated atherosclerosis; DCM, diabetic cardiomyopathy; Dox, doxorubicin; eNOS, endothelial nitric oxide synthase; ERK, extracellular regulated protein kinase; ER, endoplasmic reticulum; Foxo, forkhead box-O transcription factors; I/R, ischemia/reperfusion; JAK, Janus kinase; LDH, lactate dehydrogenase; MI, myocardial infarction; MANF, mesencephalic astrocyte-derived neurotrophic factor; MAPK, mitogen-activated protein kinase; NGF, nerve growth factor; NF-κB, nuclear factor kappa-B; NO, nitric oxide; PA, Palmitic acid; PI3K, phosphatidylinositol 3-kinase; Rot, rotenone; STAT, signal transducer and activator of transcription; Trk, tropomyosin receptor kinase; TRPV1, transient receptor potential vanilloid-1; 7,8,3′-THF, 7,8,3′-Trihydroxyflavone; 7,8-DHF, 7,8-Dihydroxyflavone.

## Data Availability

No new data were created or analyzed in this study.

## Abbreviations

BDNF	Brain-derived neurotrophic factor
7,8-DHF	7,8-Dihydroxyflavone
Rot	Rotenone
NO	Nitric oxide
eNOS	Endothelial nitric oxide synthase
DMAS	Diabetes mellitus-accelerated atherosclerosis
Trk	Tropomyosin receptor kinase
Dox	Doxorubicin
MAPK	Mitogen-activated protein kinase
ERK	Extracellular regulated protein kinase
PA	Palmitic acid
7,8,3′-THF	7,8,3′-Trihydroxyflavone
DCM	Diabetic cardiomyopathy
Foxo	Forkhead box-O transcription factors
Akt	Serine/threonine-specific protein kinase
I/R	Ischemia/reperfusion
ER	Endoplasmic reticulum
PI3K	Phosphatidylinositol 3-kinase
NGF	Nerve growth factor
LDH	Lactate dehydrogenase
CNTF	Ciliary neurotrophic factor
AAV	Adeno associated virus
MANF	Mesencephalic astrocyte-derived neurotrophic factor
JAK	Janus kinase
STAT	Signal transducer and activator of transcription
NF-κB	Nuclear factor kappa-B
CDNF	Cerebral dopamine neurotrophic factor
TRPC	Transient receptor potential canonical
Caspase	Cysteinyl aspartate specific proteinase
mTOR	Mammalian target of rapamycin
VE-cadherin	Vascular endothelial cadherin
p75^NTR^	p75 neurotrophin receptor
NT	Neurotrophin
NTF	Neurotrophic factor
GFRa	GDNF family receptor alpha
GPI	Glycosylphos-phoinositol
RET	Rearranged during Transfection
gp130	Glycoprotein 130
NPTN	Neuroplastin
HF	Heart failure
UPR	Unfolded protein response
GRP78	Glucose regulatory protein 78
CAD	Coronary artery disease
CNS	Central nervous system
MI	Myocardial infarction
LV	Left ventricle
Bcl-2	B-cell lymphoma-2
ROS	Reactive oxygen species
NADPH	Nicotinamide adenine dinucleotide phosphate hydrogen
VEGF	Vascular endothelial growth factor
GAP-43	Growth-associated protein 43
CaMKII	Calcium/calmodulin-dependent protein kinase II
NE	Norepinephrine
Ang II	Angiotensin II
HIIT	High-intensity interval training
RT	Resistance training
VSMC	Vascular smooth muscle cell
hMSCs	Human multipotent mesenchymal stromal cells
Bmal1	Brain and muscle Arnt-like protein 1
TRPV1	Transient receptor potential vanilloid-1
CGRP	Calcitonin gene-related peptide
YY1	Yin Yang 1
PGC-1α	Peroxisome proliferator-activated receptor γ coactivator 1α
SA	Stable angina
AMI	Acute myocardial infarction
AMPK	AMP-activated protein kinase
UA	Unstable angina
